# Sitting Closer to Friends than Enemies, Revisited

**DOI:** 10.1007/s00224-014-9558-4

**Published:** 2014-07-01

**Authors:** Marek Cygan, Marcin Pilipczuk, Michał Pilipczuk, Jakub Onufry Wojtaszczyk

**Affiliations:** 1 Institute of Informatics, University of Warsaw, Warsaw, Poland; 2 Department of Informatics, University of Bergen, Bergen, Norway; 3 Google Inc., Warsaw, Poland

**Keywords:** Signed graphs, Relationships, Embedding into metric space

## Abstract

Signed graphs, i.e., undirected graphs with edges labelled with a plus or minus sign, are commonly used to model relationships in social networks. Recently, Kermarrec and Thraves (2011) initiated the study of the problem of appropriately visualising the network: They asked whether any signed graph can be embedded into the metric space ${{\mathbb {R}}}^{l}$ in such a manner that every vertex is closer to all its friends (neighbours via positive edges) than to all its enemies (neighbours via negative edges). Interestingly, embeddability into ${{\mathbb {R}}}^{1}$ can be expressed as a purely combinatorial problem. In this paper we pursue a deeper study of this case, answering several questions posed by Kermarrec and Thraves. First, we refine the approach of Kermarrec and Thraves for the case of complete signed graphs by showing that the problem is closely related to the recognition of proper interval graphs. Second, we prove that the general case, whose polynomial-time tractability remained open, is in fact *NP*-complete. Finally, we provide lower and upper bounds for the time complexity of the general case: we prove that the existence of a subexponential time (in the number of vertices and edges of the input signed graph) algorithm would violate the Exponential Time Hypothesis, whereas a simple dynamic programming approach gives a running time single-exponential in the number of vertices.

## Introduction

Undirected graphs with edges labelled positively (by a + ) and negatively (by a −), called *signed graphs*, in many applications serve as a very simple model of relationships between a group of people, e.g., in a social network. Sign labels can express in a simplified way mutual relations, like staying in a relationship, family bonds or conflicts, by classifying them either as *friendship* (+ edge), *hostility* (− edge) or *ambivalence* (no edge). In particular, much effort has been put into properly understanding and representing the structure of the network, balancing it or naturally partitioning into clusters [1, 3, 6, 15–18, 21]. One of the problems is to visualize the model graph properly, i.e., in such a way that positive relations tend to make vertices be placed close to each other, while negative relations imply large distances between vertices.

In their recent work, Kermarrec and Thraves [14] formalized this problem as follows: Consider the metric space ${{\mathbb {R}}}^{l}$ with the Euclidean metric denoted by *d*. Given a signed graph *G*, is it possible to embed the vertices of *G* in ${{\mathbb {R}}}^{l}$ so that for any positive edge *uu*_1_ and negative edge *uu*_2_ it holds that *d*(*u*, *u*_1_) < *d*(*u*, *u*_2_)? This question has a natural interpretation: we would like to place a group of people so that every person is placed closer to his friends than to his enemies.

The work of Kermarrec and Thraves [14] concentrated on showing a number of examples and counterexamples for embeddability into spaces of small dimensions (1 and 2) and a deeper study of the 1-dimensional case. Interestingly enough, the case of the Euclidean line has an equivalent formulation in the language of pure combinatorics: Given a signed graph *G*, is it possible to order the vertices of *G* so that for any positive edge *uw* there is no negative edge *uv* with *v* laying between *u* and *w*? The authors made algorithmic use of this combinatorial insight: Providing the given signed graph is complete (i.e., every pair of vertices is adjacent via a positive or negative edge) they show a polynomial-time algorithm that computes an embedding into a line or reports that no such embedding exists.

Kermarrec and Thraves also posed a number of open problems in the area, including the question of the complexity of determining the embeddability of an arbitrary (not necessarily complete) graph into the Euclidean line.

### Our Results

We focus on the problem of embedding a signed graph into a line. The reformulation of the 1-dimensional case of Kermarrec and Thraves turns out to be an interesting combinatorial problem, which allows classical methods of analysis and shows interesting links with the class of proper interval graphs.

We begin with refining the result of Kermarrec and Thraves for the case of complete graphs. We prove that a complete signed graph is embeddable into a line if and only if the graph formed by the positive edges is a proper interval graph. Using this theorem one can immediately transfer all the results from the well-studied area of proper interval graphs into our setting. Most importantly, as recognition of proper interval graphs can be performed in linear-time [4], we obtain a simpler algorithm for determining the embeddability of a complete graph into a line, with a linear runtime.

We next analyse the general case. We resolve the open problem posed in [14] negatively: it is *NP*-complete to resolve whether a given signed graph can be embedded into a line. This hardness result also answers other questions of Kermarrec and Thraves [14]. For example, we infer that it is *NP*-hard to decide the smallest dimension of a Euclidean space in which the graph can be embedded, as such an algorithm could be used to test embeddability into a line.

Furthermore, we are able to show a lower bound on the time complexity of resolving embeddability into a line, under a plausible complexity assumption. We prove that obtaining an algorithm running in subexponential time (in terms of the total number of vertices and edges of the input graph) would contradict the *Exponential Time Hypothesis* [11] (see Section 2 for an exact statement). We complete the picture of the complexity of the problem by showing a dynamic programming algorithm that runs in *O* ^⋆^(2^*n*^) time, 1 matching the aforementioned lower bound up to a constant in the base of the exponent (*n* denotes the number of vertices of the input graph).

### Organisation of the Paper

In Section 2 we recall widely known notions and facts that are of further use, and provide the details of the combinatorial reformulation of the problem by Kermarrec and Thraves [14]. Section 3 is devoted to refinements in the analysis of the case of the complete signed graphs, while Section 4 describes upper and lower bounds for the complexity of the general case. Finally, in Section 5 we gather conclusions and ideas for further work.

## Preliminaries

### Basic Definitions

For a finite set *V*, by an *ordering* of *V* we mean a bijection *π* : *V* → {1, 2, …, |*V*|}. We sometimes treat an ordering *π* as a linear order on *V* and for *u*, *v* ∈ *V* we write *u*≤_*π*_ *v* to denote *π*(*u*)≤*π*(*v*). A *lexicographic ordering* imposed by *π* on pairs of elements from *V* is an ordering $\pi ^{\prime }$ of *V*×*V* defined as follows: $(a, b)\leq _{\pi ^{\prime }} (c, d)$ if and only if *a* < _*π*_ *c*, or *a* = *c* and *b*≤_*π*_ *d*. If *π* is an ordering of vertices of a directed graph *G*, then we say that *π* is a *topological ordering* if and only if for every (*v*, *w*) ∈ *E*(*G*) we have that *v*≤_*π*_ *w*. A directed graph admits a topological ordering of vertices if and only if it is acyclic.

In a graph *G* = (*V*, *E*) the *neighbourhood* of a vertex *v*, denoted *N*(*v*), is the set of all its neighbours, i.e., {*w* : *vw* ∈ *E*}. The *closed neighbourhood* of *v* is defined as $N[v]=N(v)\cup \{v\}$.

A *signed graph* is a triple *G* = (*V*, *E* ^+^, *E* ^−^), where $E^{+}, E^{-}\subseteq \binom {V}{2}$ and $E^{+}\cap E^{-}=\emptyset $. We view a signed graph as an undirected simple graph with two possible labels on the edges: positive (+ ) and negative (−). We call the edges from *E* ^+^ positive, while those from *E* ^−^ negative. The graph *G* ^+^=(*V*, *E* ^+^) is called the *positive part* of *G*, and *G* ^−^=(*V*, *E* ^−^) — the *negative part*. A signed graph is called *complete* if $E^{+}\cup E^{-}=\binom {V}{2}$, i.e., every pair of vertices is adjacent via a positive or negative edge.

### Proper Interval Graphs

Let *G* = (*V*, *E*) be an undirected graph, ${{\mathcal {I}}}$ be a family of size |*V*| of intervals on real line with nonempty interiors and pairwise different endpoints and $\iota :V\to {{\mathcal {I}}}$ be any bijection. We say that ${{\mathcal {I}}}$ is an *interval model* for *G* if for every *v*, *w* ∈ *V*, *v*≠*w*, *vw* ∈ *E* is equivalent to $\iota (v)\cap \iota (w)\neq \emptyset $. ${{\mathcal {I}}}$ is a *proper interval model* if, additionally, none of the intervals is entirely contained in any other. Graphs having an interval model are called *interval graphs*, while if a proper interval model exists as well, we call them *proper interval graphs*.

Looges and Olariu showed the following combinatorial characterization of proper interval graphs. An ordering *π* of the vertex set of a graph *G* = (*V*, *E*) is called an *umbrella ordering* if whenever *π*(*v*_1_) < *π*(*v*_2_) < *π*(*v*_3_), then *v*_1_ *v*_3_ ∈ *E* implies *v*_1_ *v*_2_ ∈ *E* and *v*_2_ *v*_3_ ∈ *E*.

### **Theorem 2.1** ([19])

*A graph G is a proper interval graph if and only if G admits an umbrella ordering.*

### Exponential Time Hypothesis

[11]: The *Exponential Time Hypothesis* (ETH for short) asserts that there exists a constant *C* > 0 such that no algorithm solving the 3-CNF-SAT problem in *O*(2^*Cn*^) exists, where *n* denotes the number of variables in the input formula.

### Combinatorial Problem Statement

In [14], Kermarrec and Thraves work with the metric definition of the problem: Given a signed graph *G* = (*V*, *E* ^+^, *E* ^−^) a feasible embedding of *G* in the Euclidean space ${{\mathbb {R}}}^{l}$ is such a function $f:V\to {{\mathbb {R}}}^{l}$ that for all *u*_1_, *u*_2_, *u*, if *u*_1_ *u* ∈ *E* ^+^ and *u*_2_ *u* ∈ *E* ^−^, then *d*(*f*(*u*_1_), *f*(*u*)) < *d*(*f*(*u*_2_), *f*(*u*)) (recall that *d* stands for the Euclidean distance in ${{\mathbb {R}}}^{l}$). However, for the 1-dimensional case they have in essence proved the following result:

### **Theorem 2.2** (Lemmata 3 and 4 of [14], rephrased)

*A signed graph G = (V, E* ^+^ *, E* ^−^ *) has a feasible embedding in a line iff there is an ordering π of V such that for every u ∈ V:*

- *(i) there are no u*_1_ *< *_*π*_ *u*_2_ *< *_*π*_ *u such that u*_1_ *u ∈ E* ^+^ *and u*_2_ *u ∈ E* ^−^;
- *(ii) there are no u*_1_ *> *_*π*_ *u*_2_ *> *_*π*_ *u such that u*_1_ *u ∈ E* ^+^ *and u*_2_ *u ∈ E* ^−^.

We will jointly call conditions *(i)* and *(ii)the condition imposed on u*. Somewhat abusing the notation, the ordering *π* will also be called *an embedding of G into the line*. Therefore, from now on we are working with the following combinatorial problem that is equivalent to the version considered by Kermarrec and Thraves: 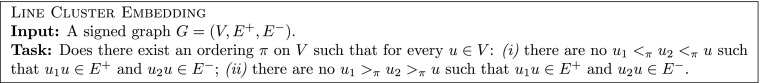


## The Complete Signed Graph Case

In their work, Kermarrec and Thraves [14] announced a polynomial-time algorithm solving the Line Cluster Embedding problem in the case where the input signed graph is complete. Their line of reasoning was essentially as follows: if a signed graph can be embedded into a line, then its positive part has to be chordal. However, for a connected chordal graph with at least 4 vertices that actually is embeddable into a line, every *perfect elimination ordering* of the graph is a feasible solution. Therefore, having checked that the graph is chordal and computed a perfect elimination ordering of every connected component, we can simply verify whether the obtained ordering is a correct line embedding.

**Fig. 1:**
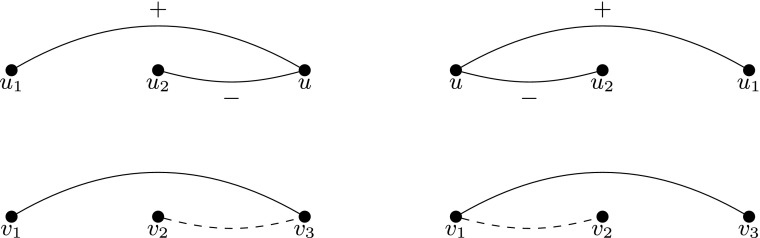
A picture proof of Lemma 3.1. The *first row* presents forbidden situations in a feasible embedding of *G* into a line, whereas the *second row* presents forbidden situations in an umbrella ordering

We refine the approach of Kermarrec and Thraves by showing the following simple observation (see also Fig. 1 for an illustration).

### **Lemma 3.1**

*For a signed complete graph G = (V, E* ^+^ *, E* ^−^ *), an ordering π of V is a feasible embedding of G into a line if and only if π is an umbrella ordering of the positive part of G.*

### *Proof*

If *π* is not a feasible embedding of *G* into a line, then there exists *u*_1_, *u*_2_, *u* ∈ *V* such that *u*_1_ *u* ∈ *E* ^+^, *u*_2_ *u* ∈ *E* ^−^, and *u*_2_ lies between *u*_1_ and *u* in the ordering *π*. Consequently, *u*_1_ *u* is an edge of the positive part, but *u*_2_ *u* is not, hence *π* is not an umbrella ordering of *G* ^+^.

In the other direction, if *π* is not an umbrella ordering of *G* ^+^, then there exist *v*_1_ < _*π*_ *v*_2_ < _*π*_ *v*_3_ such that *v*_1_ *v*_2_ ∈ *E* ^+^ but *v*_1_ *v*_3_ ∉ *E* ^+^ or *v*_3_ *v*_2_ ∉ *E* ^+^. As *G* is a complete graph, *v*_1_ *v*_3_ ∈ *E* ^−^ or *v*_3_ *v*_2_ ∈ *E* ^−^. In the first case, we have a violation of condition (ii) imposed on *v*_1_, and in the second case we have a violation of condition (i) imposed on *v*_3_. Consequently, *π* is not a feasible embedding of *G* into a line.

### **Corollary 3.2**

*A complete signed graph G = (V, E* ^+^ *, E* ^−^ *) is embeddible in* ${{\mathbb {R}}}^{1}$ *if and only if G* ^+^ *=(V, E* ^+^ *) is a proper interval graph.*

Recall that proper interval graphs are a subclass of chordal graphs; therefore, the result nicely fits into the picture of Kermarrec and Thraves. Moreover, the theory of proper interval graphs is well-studied, so many results from that area can be immediately translated to our setting. For instance, many NP-complete problems become solvable in polynomial time on proper interval graphs (e.g., [2, 10, 13, 20]), and the linear-time algorithm of Corneil et al. [4] for recognizing proper interval graphs immediately solves the Line Cluster Embedding problem in linear time in case of a complete signed graph.

### **Corollary 3.3**

*Assuming the input graph is complete and given as the set of positive edges,* Line Cluster Embedding *can be solved in O(|V|+|E* ^+^ *|) time complexity. Moreover, the algorithm can produce a feasible ordering of the vertices in the same time, if such an ordering exists.*

## The General Case

### *NP*-Completeness of the General Case

In [14] Kermarrec and Thraves asked whether the Line Cluster Embedding problem is also polynomial-time solvable in the case where the input is not restricted to complete graphs. In this section we show that this is unlikely: in fact, the problem becomes *NP*-complete.

In the proof we use an auxiliary problem, called Acyclic Partition. 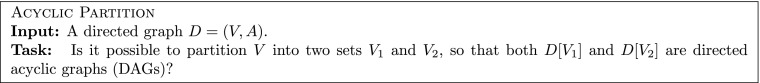


Acyclic Partition has been introduced and proven to be *NP*-complete by Eppstein and Mumford [7] and, independently, by Guillemot et al. [9]. In both these papers it was used as a pivot problem for proving *NP*-completeness of other problems. For the sake of completeness we would like to revisit the *NP*-hardness proof, because we need explicit bounds on the size of the directed graph obtained in the reduction for further applications. We begin with an instance of the Set Splitting problem, which is known to be *NP*-complete [8]. 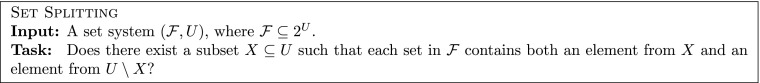


#### **Lemma 4.1**

*There exists a polynomial-time algorithm that given an instance* $({{\mathcal {F}}}, U)$ *of* Set Splitting *outputs an equivalent instance G = (V, A) of* Acyclic Partition, *for which* $|V|=|U|+{\sum }_{F\in {{\mathcal {F}}}} |F|$ *and* $|A|=3{\sum }_{F\in {{\mathcal {F}}}} |F|$.

#### *Proof*

We construct the directed graph *D* = (*V*, *A*) as follows. For every set $F\in {{\mathcal {F}}}$ and every *u* ∈ *F* we build a vertex ${c_{u}^{F}}$ and connect all the vertices corresponding to the same set *F* into a directed cycle in any order. For every element *u* ∈ *U* we build a vertex *d*_*u*_ and for every vertex of the form ${c_{u}^{F}}$ we introduce two arcs: $(d_{u}, {c_{u}^{F}})$ and $({c_{u}^{F}}, d_{u})$. This concludes the construction; it is easy to verify the claimed sizes of *V* and *A*.

Let us formally prove that the instances are equivalent. Let *X* be any solution to the $({{\mathcal {F}}}, U)$ instance of Set Splitting. Let $V_{1}=\{d_{u}:u\in X\}\cup \{{c_{u}^{F}}:u\in U\setminus X\}$ and $V_{2}=\{d_{u}:u\in U\setminus X\}\cup \{{c_{u}^{F}}:u\in X\}$. As *X* splits every set $F\in {{\mathcal {F}}}$, none of the cycles formed by vertices ${c_{u}^{F}}$ for fixed *F* is entirely contained in either *V*_1_ or *V*_2_. Also, for every element *u* the vertex *d*_*u*_ becomes isolated in the corresponding graph *D*[*V*_*i*_], as all his neighbours belong to *V*_3−*i*_. Therefore, both *D*[*V*_1_] and *D*[*V*_2_] are collections of isolated vertices and directed paths and (*V*_1_, *V*_2_) is a solution to the Acyclic Partition instance.

In the other direction, let (*V*_1_, *V*_2_) be a solution to the instance of Acyclic Partition. Let $X=\{u:d_{u}\in V_{1}\}\subseteq U$; we claim that *X* is a solution to the instance of Set Splitting. Note that, due to the 2-cycles on the vertices *d*_*u*_ and ${c_{u}^{F}}$, we have that for every *u* ∈ *X*, all vertices ${c_{u}^{F}}$ belong to *V*_2_, whereas for every *u*∉*X*, all vertices ${c_{u}^{F}}$ belong to *V*_1_.

Take any $F\in {{\mathcal {F}}}$. As the cycle formed by vertices ${c_{u}^{F}}$ is not entirely contained in any of the graphs *D*[*V*_1_], *D*[*V*_2_], there exist some *u*_1_ such that $c_{u_{1}}^{F}\in V_{1}$ and *u*_2_ such that $c_{u_{2}}^{F}\in V_{2}$. As the cycles formed by pairs $\{d_{u_{1}}, c_{u_{1}}^{F}\}$ and $\{d_{u_{2}}, c_{u_{2}}^{F}\}$ are also not entirely contained in *D*[*V*_1_] nor in *D*[*V*_2_], $d_{u_{1}}\in V_{2}$ and $d_{u_{2}}\in V_{1}$. Consequently, *u*_1_ ∈ *U*∖*X*, *u*_2_ ∈ *X* and *F* is split.

#### **Lemma 4.2**

*There exists a polynomial-time algorithm that given an instance D=(V, A) of* Acyclic Partition *outputs an equivalent instance* $H=(V^{\prime }, E^{+}, E^{-})$ *of* Line Cluster Embedding, *such that* $|V^{\prime }|=|V|+|A|+1$ *, |E* ^+^ *|=2|A| and |E* ^−^ *|=|A|+|V|.*

#### *Proof*

We construct the graph *H* as follows: The set of vertices, $V^{\prime }$, consists of:

- a *special* vertex *s*;
- for every *e* ∈ *A*, a *checker* vertex *c*_*e*_;
- for every *v* ∈ *V*, an *alignment* vertex *a*_*v*_.

We construct the edges of the signed graph as follows:

- for every *e* ∈ *A*, we introduce a positive edge *sc*_*e*_;
- for every *v* ∈ *V*, we introduce a negative edge *sa*_*v*_;
- for every arc (*v*, *w*) ∈ *A*, we introduce a positive edge *c*_(*v*, *w*)_ *a*_*v*_ and a negative edge *c*_(*v*, *w*)_ *a*_*w*_.

This concludes the construction; it is easy to verify the claimed sizes of $V^{\prime }, E^{+}, E^{-}$.

Let us prove equivalence of the instances. Let *π*, an ordering of $V^{\prime }$, be a solution of the Line Cluster Embedding instance $(V^{\prime }, E^{+}, E^{-})$. As the special vertex *s* is adjacent via positive edges to all the checker vertices, and via negative edges to all the other, alignment, vertices, in the ordering *π* the checker vertices together with the special vertex have to form an interval, i.e., a set of consecutive elements with respect to *π*. Let *V*_1_ be the set of those *v* ∈ *V* for which *a*_*v*_ is to the left of this interval, whereas *V*_2_ is the set of those *v* ∈ *V* for which *a*_*v*_ is to the right of this interval. Formally, *V*_1_={*v* ∈ *V* : *a*_*v*_≤_*π*_ *s*} and *V*_2_={*v* ∈ *V* : *a*_*v*_≥_*π*_ *s*}. We claim that (*V*_1_, *V*_2_) is a feasible solution of the Acyclic Partition instance (*V*, *A*). Consider any arc (*v*, *w*) such that *v*, *w* ∈ *V*_1_. As *a*_*v*_≤_*π*_ *c*_(*v*, *w*)_, *a*_*w*_≤_*π*_ *c*_(*v*, *w*)_, *c*_(*v*, *w*)_ *a*_*v*_ ∈ *E* ^+^ and *c*_(*v*, *w*)_ *a*_*w*_ ∈ *E* ^−^, then it follows that *a*_*w*_≤_*π*_ *a*_*v*_. Thus, *π* has to induce a reverse topological ordering on the vertices of *D*[*V*_1_] and, therefore, *D*[*V*_1_] has to be acyclic. Symmetrically, *D*[*V*_2_] has to be acyclic as well, which concludes the proof of (*V*_1_, *V*_2_) being a feasible solution.

Now take any solution (*V*_1_, *V*_2_) of Acyclic Partition instance (*V*, *A*). Let *π*_1_ be any topological ordering of *D*[*V*_1_] and *π*_2_ be any topological ordering of *D*[*V*_2_], by which we mean that if (*u*, *v*) is an arc of *D*[*V*_1_], *π*_1_(*u*) < *π*_1_(*v*), and the same holds for *π*_2_. Let us construct an ordering *π* of $V^{\prime }$ as follows:

- first, place all the vertices *a*_*v*_ for *v* ∈ *V*_1_ in the reverse order induced by *π*_1_;
- then, place all the checker vertices *c*_(*v*, *w*)_ for which *v* ∈ *V*_1_ and *w* ∈ *V*_2_, in any order;
- then, place all the checker vertices *c*_(*v*, *w*)_ for which *v*, *w* ∈ *V*_1_, in reverse lexicographic order imposed by *π*_1_ on pairs (*v*, *w*);
- then, place the special vertex *s*;
- then, place all the checker vertices *c*_(*v*, *w*)_ for which *v*, *w* ∈ *V*_2_, in lexicographic order imposed by *π*_2_ on pairs (*v*, *w*);
- then, place all the checker vertices *c*_(*v*, *w*)_ for which *v* ∈ *V*_2_ and *w* ∈ *V*_1_, in any order;
- finally, place all the vertices *a*_*v*_ for *v* ∈ *V*_2_ in the order induced by *π*_2_.

We claim that such *π* is a feasible solution to Line Cluster Embedding instance $(V^{\prime }, E^{+}, E^{-})$.

Note that the positive neighbours of the special vertex *s* form an interval, therefore the condition imposed on this vertex is satisfied. Now consider a checker vertex *c*_(*v*, *w*)_. If *v*, *w* belong to different sets *V*_1_, *V*_2_, then the only negative neighbour of *c*_(*v*, *w*)_ is the first or the last of his closed neighbourhood with respect to *π*, thus satisfying the condition imposed on *c*_(*v*, *w*)_. In case when *v*, *w* ∈ *V*_1_ or *v*, *w* ∈ *V*_2_ this is also true, due to *π*_1_, *π*_2_ being topological orderings of *D*[*V*_1_], *D*[*V*_2_] respectively.

Now take any vertex *a*_*v*_, by symmetry assume *v* ∈ *V*_1_. We need to prove that the condition imposed on *a*_*v*_ is satisfied as well. The neighbours of *v* consist of:

1. positive neighbours $c_{(v, v^{\prime })}$, such that $v^{\prime }\in V_{2}$;
2. positive neighbours $c_{(v, v^{\prime })}$, such that $v^{\prime }\in V_{1}$;
3. negative neighbours $c_{(v^{\prime }, v)}$, such that $v^{\prime }\in V_{1}$;
4. negative neighbours $c_{(v^{\prime }, v)}$, such that $v^{\prime }\in V_{2}$.

We now verify that by the construction of *π* the neighbours of *a*_*v*_ lie in this very order with respect to *π*. Clearly, the order in which we placed the checkers in *π* ensures that the neighbours from (1) are placed before the neighbours from (2) and that the neighbours from (3) are placed before the neighbours from (4). Thus the only non-trivial check is whether the vertices from (2) lie before the vertices from (3). Assume otherwise, that there are some $v_{1}^{\prime }, v_{2}^{\prime }$ such that $(v, v_{1}^{\prime })\in A$, $(v_{2}^{\prime }, v)\in A$, but $c_{(v, v_{1}^{\prime })}>_{\pi }c_{(v_{2}^{\prime }, v)}$. Then $v_{2}^{\prime }<_{\pi _{1}}v$ as *π*_1_ is a topological ordering of *D*[*V*_1_], so the pair $(v_{2}^{\prime }, v)$ is lexicographically smaller than the pair $(v, v_{1}^{\prime })$. Hence $c_{(v, v_{1}^{\prime })}>_{\pi }c_{(v_{2}^{\prime }, v)}$, a contradiction with the construction of *π*.

We have verified that for all the vertices the conditions imposed on them are satisfied, so the instances are equivalent.

The *NP*-completeness of the Set Splitting problem [8], together with Lemmata 4.1, 4.2 and a trivial observation that Line Cluster Embedding is in *NP*, gives us the following theorem.

#### **Theorem 4.3**

*The* Line Cluster Embedding *problem is NP-complete.*

As mentioned before, the question of finding the smallest dimension of the Euclidean space, into which the given graph can be embedded, clearly generalizes testing embeddability into a line. Therefore, we have the followingcorollary.

#### **Corollary 4.4**

*It is NP-hard to decide the smallest dimension of the Euclidean space, into which a given signed graph can be embedded.*

### Lower Bound on the Complexity

In this subsection we observe that the presented chain of reductions enables us also to establish a lower bound on the complexity of solving Line Cluster Embedding under ETH. Firstly, let us complete the chain of the reductions.

#### **Lemma 4.5**

*There exists a polynomial-time algorithm that given an instance φ of 3* -CNF-SAT *with n variables and m clauses, outputs an equivalent instance* $(U, {\mathcal {F}})$ *of* Set Splitting *with |U|=2n+1 and* ${\sum }_{F\in {\mathcal {F}}} |F|=2n+4m$.

#### *Proof*

We construct the instance $(U, {{\mathcal {F}}})$ as follows. The universe *U* consists of one special element *s* and two literals *x*, ¬*x* for every variable *x* of *φ*. The family ${\mathcal {F}}$ includes *(a)* for every variable *x*, a set *F*_*x*_={*x*, ¬*x*}; *(b)* for every clause *C*, a set *F*_*C*_ consisting of *s* and all the literals in *C*. It is easy to check the claimed sizes of $U, {\mathcal {F}}$. We claim that the instance of Set Splitting $(U, {\mathcal {F}})$ is equivalent to the instance *φ* of 3-CNF-SAT.

Assume that *ψ* is a boolean evaluation of variables of *φ* that satisfies *φ*. We construct a set $X\subseteq U$ as follows: *X* consists of all the literals that are true in *ψ*. Now, every set *F*_*x*_ is split, as exactly one of the literals is true and one is false, whereas every set *F*_*C*_ is split as well, as it contains a true literal, which belongs to *X*, and the special element *s*, which does not.

Now assume that $X\subseteq U$ is a solution to the Set Splitting instance $(U, {{\mathcal {F}}})$. As taking *U*∖*X* instead of *X* also yields a solution, without losing generality we can assume that *s*∉*X*. Every set *F*_*x*_ is split by *X*; therefore, exactly one literal of every variable belongs to *X* and exactly one does not. Let *ψ* be a boolean evaluation of variables of *φ* such that it satisfies all the literals belonging to *X*. Observe that *ψ* satisfies *φ* : for every clause *C* the set *F*_*C*_ has to be split, so, as *s*∉*X*, one of the literals of *C* belongs to *X* and, thus, is satisfied by *ψ*.

Note that by pipelining Lemmata 4.5, 4.1 and 4.2, we obtain a reduction from 3-CNF-SAT to Line Cluster Embedding, where the output instance has a number of vertices and edges bounded linearly in the number of variables and clauses of the input formula. As the Exponential Time Hypothesis also excludes a possibility of having an algorithm for 3-CNF-SAT with running time subexponential in the total number of variables and clauses of the formula [12], we obtain the following.

#### **Theorem 4.6**

*Unless ETH fails, there is a constant δ>0 such that there is no algorithm that given a (V, E* ^+^ *, E* ^−^ *) instance of* Line Cluster Embedding *problem, solves it in* $O(2^{\delta (|V|+|E^{+}|+|E^{-}|)})$ *time.*

### A Single-Exponential Algorithm for Line Cluster Embedding

Note that the trivial brute-force algorithm for Line Cluster Embedding checks all possible orderings, working in *O* ^⋆^(*n*!) time. To complete the picture of the complexity of Line Cluster Embedding, we show that a simple dynamic programming approach can give single-exponential time complexity. This matches the lower bound obtained from under Exponential Time Hypothesis (up to a constant in the base of the exponent).

Before we proceed with the description of the algorithm, let us state a combinatorial observation that will be its main ingredient. Let (*V*, *E* ^+^, *E* ^−^) be the given Line Cluster Embedding instance. For $X\subseteq V$ and *v*∉*X* we will say that *v* is *good* for the set *X* iff

- no vertex *w* ∈ *X* that is adjacent to *v* via a negative edge is simultaneously adjacent to some vertex from $V\setminus (X\cup \{v\})$ via a positive edge;
- no vertex $w\in V\setminus (X\cup \{v\})$ that is adjacent to *v* via a negative edge is simultaneously adjacent to some vertex from *X* via a positive edge.

#### **Lemma 4.7**

*An ordering π is a feasible solution of (V, E* ^+^ *, E* ^−^ *) if and only if every vertex v ∈ V is good for the set {u : u < *_*π*_ *v}.*

#### *Proof*

One direction is obvious: if *π* is a feasible solution, then every vertex *v* has to be good for the set {*u* : *u* < _*π*_ *v*}. If *v* would not be good for {*u* : *u* < _*π*_ *v*}, there would exist a vertex *w* certifying that *v* is not good, and the condition imposed upon *w* would be not satisfied.

Now assume that every vertex *v* ∈ *V* is good for {*u* : *u* < _*π*_ *v*} and take an arbitrary vertex *v* ∈ *V*. If there were vertices *u*_1_ < _*π*_ *u*_2_ < _*π*_ *v* such that *u*_1_ *v* ∈ *E* ^+^ while *u*_2_ *v* ∈ *E* ^−^, then *u*_2_ would not be good for the set {*u* : *u* < _*π*_ *u*_2_}, a contradiction. Similarly, if there were vertices *u*_1_ > _*π*_ *u*_2_ > _*π*_ *v* such that *u*_1_ *v* ∈ *E* ^+^ while *u*_2_ *v* ∈ *E* ^−^, then *u*_2_ would not be good for the set {*u* : *u* < _*π*_ *u*_2_}, a contradiction as well. Therefore, the condition imposed on *v* is satisfied for an arbitrary choice of *v*.

#### **Theorem 4.8**

Line Cluster Embedding *can be solved in O* ^*⋆*^ *(2* ^*n*^ *) time and space complexity. Moreover, the algorithm can also output a feasible ordering of the vertices, if it exists.*

#### *Proof*

Let (*V*, *E* ^+^, *E* ^−^) be the given Line Cluster Embedding instance. Let *W* = {(*v*, *X*):*v* is good for *X*}. Let us construct a directed graph *D* = (*W*, *F*), where $((v, X), (v^{\prime }, X^{\prime }))\in F$ if and only if $X^{\prime }=X\cup \{v\}$. As recognizing being good is clearly a polynomial time operation, the graph *D* can be constructed in *O* ^⋆^(2^*n*^) time and has that many vertices and edges. Observe that by Lemma 4.7 there is a feasible ordering *π* if and only if some sink (*v*, *V*∖{*v*}) is reachable from some source (*u*, *∅*); indeed, such a path corresponds to introducing the vertices of *V* one by one in such a manner that each of them is good for the respective prefix. Reachability of any sink from any source can be, however, tested in time linear in the size of the graph using a breadth-first search. The search can also reconstruct the path in the same complexity, thus constructing the feasible solution.

## Conclusions

In this paper we addressed a number of problems raised by Kermarrec and Thraves in [14] for embeddability of a signed graph into a line. We refined their study of the case of a complete signed graph by showing relation with proper interval graphs. Moreover, we have proven *NP*-hardness of the general case and shown an almost complete picture of its complexity.

Although the general case of the problem appears to be hard, real-life social networks have a certain structure. Is it possible to develop faster, maybe even polynomial-time algorithms for classes of graphs reflecting this structure?

## Footnotes

1 The *O* ^⋆^() notation suppresses factors that are polynomial in the input size.

## References

[1] Antal T, Krapivsky PL, Redner S (2005). Dynamics of social balance on networks. Phys. Rev. E.

[2] Belmonte, R., Vatshelle, M.: Graph classes with structured neighborhoods and algorithmic applications. In: Kolman, P., Kratochvíl, J. (eds.), WG, volume 6986 of Lecture Notes in Computer Science, pp. 47–58. Springer (2011)

[3] Cartwright D, Harary F (1956). Structural balance: a generalization of heider’s theory. Psychol Rev.

[4] Corneil DG, Kim H, Natarajan S, Olariu S, Sprague AP (1995). Simple linear time recognition of unit interval graphs. Inf. Process. Lett..

[5] Cygan, M., Pilipczuk, M., Pilipczuk, M., Wojtaszczyk, J.O.: Sitting closer to friends than enemies, revisited. In: Rovan, B., Sassone, V., Widmayer, P. (eds.), MFCS, volume 7464 of Lecture Notes in Computer Science, pp. 296–307. Springer (2012)

[6] Davis JA (1967). Clustering and structural balance in graphs. Hum. Relat..

[7] Eppstein, D., Mumford, E.: Self-overlapping curves revisited. In: Mathieu, C. (ed.), SODA, pp. 160–169. SIAM (2009)

[8] Garey MR, Johnson DS (1979). Computers and Intractability: A Guide to the Theory of NP-Completeness.

[9] Guillemot S, Jansson J, Sung W-K (2011). Computing a smallest multilabeled phylogenetic tree from rooted triplets. IEEE/ACM Trans. Comput. Biol. Bioinform..

[10] Ibarra L (2009). A simple algorithm to find hamiltonian cycles in proper interval graphs. Inf. Process. Lett..

[11] Impagliazzo R, Paturi R (2001). On the complexity of k-SAT. J. Comput. Syst. Sci..

[12] Impagliazzo R, Paturi R, Zane F (2001). Which problems have strongly exponential complexity. J. Comput. Syst. Sci..

[13] Ioannidou, K., Mertzios, G.B., Nikolopoulos, S.D.: The longest path problem is polynomial on interval graphs. In: Královic, R., Niwinski, D. (eds.), MFCS, volume 5734 of Lecture Notes in Computer Science, pp. 403–414. Springer (2009)

[14] Kermarrec, A.-M., Thraves, C.: Can everybody sit closer to their friends than their enemies? In: Murlak, F., Sankowski, P. (eds.) MFCS, volume 6907 of Lecture Notes in Computer Science, pp. 388–399. Springer (2011)

[15] Kunegis, J., Schmidt, S., Lommatzsch, A., Lerner, J., Luca, E.W. De, Albayrak, S.: Spectral analysis of signed graphs for clustering, prediction and visualization. In: SDM, p. 559. SIAM (2010)

[16] Leskovec, J., Huttenlocher, D.P., Kleinberg, J.M.: Governance in social media: A case study of the wikipedia promotion process. In: Cohen, W.W., Gosling, S. (eds.) ICWSM. The AAAI Press (2010)

[17] Leskovec, J., Huttenlocher, D.P., Kleinberg, J.M.: Predicting positive and negative links in online social networks. In: Rappa, M., Jones, P., Freire, J., Chakrabarti, S. (eds.), WWW, pp. 641–650. ACM (2010)

[18] Leskovec, J., Huttenlocher, D.P., Kleinberg, J.M.: Signed networks in social media. In: Mynatt, E.D., Schoner, D., Fitzpatrick, G., Hudson, S.E., Edwards, W.K., Rodden, T. (eds.), CHI, pp. 1361–1370. ACM (2010)

[19] Looges PJ, Olariu S (1993). Optimal greedy algorithms for indifference graphs. Comput. Math. Appl..

[20] George B (2006). Mertzios. A polynomial algorithm for the k-cluster problem on the interval graphs. Electron. Notes Discret. Math..

[21] Szell M, Lambiotte R, Thurner S (2010). Multirelational organization of large-scale social networks in an online world. PNAS.

